# Alcohol’s Effects on the Lung and Lung Disease

**DOI:** 10.35946/arcr.v45.1.11

**Published:** 2025-11-21

**Authors:** Derrick R. Samuelson

**Affiliations:** Department of Internal Medicine, Division of Pulmonary, Critical Care & Sleep, University of Nebraska Medical Center, Omaha, Nebraska Nebraska Food for Health Center, University of Nebraska, Lincoln, Lincoln, Nebraska

**Keywords:** alcohol, alcohol use disorder, lung, lung disease, pneumonia, lung injury, respiratory system, pulmonary system

## Abstract

**PURPOSE:**

Alcohol misuse is widely accepted as an independent risk factor for a wide variety of lung diseases, such as pneumonia and acute respiratory distress syndrome. Alcohol induces changes in the regulatory mechanisms of the lung, both at a mechanical and immunological level. Understanding these changes might help discover new targets for drugs and therapeutic approaches for the prevention of respiratory disease following alcohol misuse.

**SEARCH METHODS:**

A systematic literature search was conducted on January 25, 2025, in PubMed, Medline, and Embase of manuscripts published between January 2000 and January 2025 using the terms (“alcohol” or “ethanol”) AND (“lung,” or “respiratory,” or “pulmonary”) AND (“pneumonia” or “damage” or “leak”). Eligible manuscripts included studies that discussed the effects of ethanol on the lungs.

**SEARCH RESULTS:**

A total of 962 publications were identified; after excluding duplicates and research not covering alcohol-related lung effects (e.g., studies investigating liver damage or alcohol-related tissue injury; 814 articles), 148 studies were reviewed. An additional 15 papers from before 2000 were included as historical precedents for the current research cited. Of the 148 studies, 114 were cited in this review; previous review articles and those discussing in utero or prenatal alcohol exposure were excluded (34 articles).

**DISCUSSION AND CONCLUSIONS:**

The lungs are particularly susceptible to infections and injury following alcohol misuse. Several key mechanisms by which alcohol misuse drives lung damage have been identified. Alcohol misuse leads to impaired mucus-facilitated clearance of bacterial pathogens, increases the aspiration of microbes from the upper alimentary tract, and suppresses tissue recruitment and function of innate and adaptive immune cells. Alcohol-related reductions in antioxidant levels, trace metals, and metabolites may also contribute to lung disease in people with underlying alcohol misuse. Several regulatory molecules may play crucial roles in alcohol-induced disease processes. Although there are currently no approved therapies to combat the detrimental effects of chronic alcohol consumption on the respiratory system, these molecules may be potential therapeutic targets to guide future investigation. Despite these advancements, limitations and knowledge gaps in the field still exist. For example, few studies have investigated dose- and duration-dependent effects of alcohol on the lung, sex-specific differences in lung responses, and the interaction of alcohol with other coexposures/comorbidities, such as smoking and HIV. In addition, well-defined observational and longitudinal human studies employing robust measures of alcohol use are limited. These gaps represent novel opportunities for more thorough and robust experimental designs of human and animal studies investigating alcohol-associated lung disease.

**KEY TAKEAWAYS**
- Chronic alcohol consumption disrupts the mechanical functions of the lungs, leading to impaired mucus-facilitated clearance, increased aspiration, and impairment of the alveolar epithelium barrier.
- Alcohol misuse suppresses tissue recruitment of neutrophils, impairs alveolar macrophages, and decreases the number of dendritic cells and circulating lymphocytes, all of which increase susceptibility to respiratory infection.
- Alcohol-related reductions in antioxidant levels, trace metals, and metabolites may also contribute to lung disease in people with underlying alcohol misuse.
- Alcohol misuse significantly increases the risk of developing community-acquired pneumonia, with the relative risk increasing for every 10 to 20 grams of alcohol consumed per day.
- Alcohol misuse is also an independent risk factor for the development of acute respiratory distress syndrome (ARDS), with a twofold increase in risk of developing ARDS following alcohol misuse.

Alcohol intoxication (drinking an excess of alcohol at one time) is well described in numerous written records; however, the effects of alcohol on lung health have only been recently recognized. One of the first recorded descriptions of alcohol’s effects on lung health occurred in the late 18th century, when Benjamin Rush noted that excessive alcohol consumption was associated with pneumonia.^1^ Subsequently, nearly a century passed before William Osler reinvigorated the investigation of alcohol and the lung, noting that alcohol misuse was the most important risk factor for pneumonia.^2^ Since these key early observations, it has become widely accepted that alcohol use disorder (AUD) is an independent risk factor for a wide variety of lung infections, including bacterial pneumonias and tuberculosis, and for increased morbidity and mortality.^3^

Alcohol increases the risk of pneumonia via the impairment of several critical host defense mechanisms. First, chronic alcohol misuse significantly impairs mucus-facilitated clearance of bacterial pathogens from the upper airway by impairing the ability of airway cilia to speed up in response to stimulation, which in turn decreases mucociliary clearance.^4^ Alcohol misuse also increases the aspiration of microbes from the upper alimentary tract, which, in combination with impaired mucociliary clearance, increases the risk of pathogenic bacteria entering the lungs. Chronic alcohol consumption also significantly affects both innate and adaptive immune responses. It suppresses tissue recruitment of neutrophils during infection and inflammation,^5^ impairs the pathogen-clearing and immune responses of alveolar macrophages,^6^–^12^ decreases the number of dendritic cells and interferes with their differentiation and function,^13^–^15^ and decreases the number and function of circulating and recruited lymphocytes.^9^,^12^

A significant and independent increase in the risk of acute respiratory distress syndrome (ARDS) has likewise become a widely accepted pathological consequence of alcohol misuse. ARDS is a severe form of acute lung injury that has a mortality rate of 30% to 50%, even with state-of-the-art modern medical care in an intensive care unit.^16^ AUD independently conferred an approximately twofold increase in risk of developing ARDS.^17^ Further studies have shown that the risk of ARDS in patients with severe sepsis was closer to fourfold higher in those with underlying AUD; this risk increase was independent of factors, such as age, smoking, severity of illness, and nutritional status.^18^ Taken together, these findings indicate that drinking patterns that define AUD are associated with a significantly increased risk of serious lung infections and acute lung injury (Figure 1). The impacts of alcohol on the lungs are multi-factorial (Table 1), and this review will discuss key aspects of alcohol’s effects on lung health.

## Search Methods Employed

Three databases (PubMed, Medline, and Embase) were searched on January 25, 2025, for manuscripts published between January 2000 and January 2025, using the terms (“alcohol” or “ethanol”) AND (“lung,” or “respiratory,” or “pulmonary”) AND (“pneumonia” or “damage” or “leak”) in the title or abstract. Articles published in this time frame were included.

## Results of the Literature Search

A total of 962 publications were identified in the databases during this search. After excluding duplicates and research not covering alcohol-related lung effects (e.g., studies investigating liver damage or other alcohol-related tissue injury; 814 articles), 148 studies were reviewed. An additional 15 papers from before 2000 were included as historical precedents for the current research cited. Of the 148 studies, 114 were cited in this review; previous review articles and those discussing in utero or prenatal alcohol exposure were excluded (34 articles). Eligible manuscripts included studies that discussed the impact of ethanol on the lungs and lung disease.

The following sections briefly review the effects of alcohol misuse on the lungs, incorporating insights gathered from the literature search to provide context. Throughout the review, the terms “alcohol” and “alcohol-associated lung disease” are used for human studies and general conclusions, and “ethanol” and “ethanol-induced lung disease” apply to animal studies (typically in rodents). Chronic ethanol feeding in rodents typically refers to either the Lieber-DeCarli diet^19^ (a liquid diet that provides up to about one-third of the calories from ethanol) or the National Institute on Alcohol Abuse and Alcoholism (NIAAA) model diet^20^ (a 10-day Lieber-DeCarli diet combined with a single binge of 5 g ethanol per kilogram body weight). Other ethanol feeding models, such as ethanol in the drinking water, were also used in some of the cited studies; however, these models were less common.

## Results of the Studies Reviewed

### Alcohol-Related Mechanisms of Lung Injury

Alcohol misuse can negatively impact lung physiology, thereby enhancing susceptibility to infection and injury. As discussed in the following sections, alcohol may induce such injuries through several mechanisms, including alterations in the lung epithelial barrier, reduced ability of lung cells to remove foreign materials (mucociliary clearance), and impairment of the defensive immune response.

#### Effects on lung epithelial barrier

Chronic ethanol ingestion for as little as 6 weeks in rodents renders the lung susceptible to acute edematous injury (i.e., a sudden onset of swelling caused by excess fluid trapped in the tissue).^21^ Chronic ethanol ingestion is also associated with lasting defects in the ability of the alveolar epithelium to maintain a physical barrier. Thus, primary alveolar epithelial cells isolated from ethanol-fed animals exhibited increased permeability in monolayer culture systems, as measured by a marked increase in permeability to large proteins.^22^ Animal models further suggested that ethanol interferes with the expression and formation of tight junction complexes (i.e., multi-protein complexes that form a barrier between adjacent cells and prevent leakage) within the alveolar epithelium.^23^ Ethanol consumption also caused an increase in salt and water transport across the epithelium via apical sodium channels and basolateral protein pumps (i.e., sodium-potassium adenosine triphosphatase [Na/K-ATPase] complexes). Both of these transport mechanisms were increased in the alveolar epithelium of ethanol-fed animals, likely as a compensatory response to any fluid imbalances.^22^,^24^ Additionally, the lungs of ethanol-exposed animals had decreased net vectorial fluid transport (i.e., reduced movement of specific solutes or water from one compartment to another) in vivo compared with animals fed a control diet, despite the compensatory increase in epithelial sodium channel and Na/K-ATPase function. This observation reflects a severe permeability defect in the paracellular barrier mechanisms.^22^

More recent research found that compared with control-fed mice, ethanol-fed animals exhibited significantly compromised alveolar epithelial and vascular barriers in the lungs when challenged with lipopolysaccharide (LPS) via intratracheal administration—a well-validated model of infectious or injurious insult to the lungs.^25^ Mechanistic studies investigating the effect of ethanol on lung barrier function have suggested that fibroblast-derived transforming growth factor beta (TGF-beta1) and granulocyte-macrophage colony-stimulating factor (GM-CSF) signaling are major drivers of lung barrier dysfunction. Specifically, ethanol exposure of lung fibroblasts significantly increased barrier dysfunction in naive airway epithelial cells, via paracrine activation of TGF-beta1 signaling and suppression of GM-CSF.^26^

Additionally, human bronchial epithelial BEAS-2B cells treated with the ethanol metabolite acetaldehyde exhibited marked increases in acetaldehyde-adducts with histone proteins, which was associated with a downregulation of acetylation at histones H3 and H4.^27^ This downregulation may be due, in part, not only to the formation of histone adducts but also to the decrease in the expression of histone acetyltransferases. Moreover, acetaldehyde treatment altered chromatin structure by increasing chromatin accessibility and decreasing nucleosome occupancy.^27^ However, the effects of acetaldehyde adducts on epithelial barrier function were not evaluated and may represent another mechanism by which ethanol alters barrier function in the lungs.

The effects of alcohol misuse on human lung epithelial barrier function are ill-defined. Several studies demonstrated that people with AUD have impaired permeability of capillaries in the alveoli at baseline and develop more extensive pulmonary edema in the setting of ARDS compared with people without AUD.^28^,^29^ More recently, bronchial epithelial cells obtained from individuals with AUD showed significantly decreased barrier function after infection with severe acute respiratory syndrome coronavirus 2 (SARS-CoV-2). In contrast, the barrier function of bronchial epithelial cells obtained from individuals without AUD was enhanced after SARS-CoV-2 infection. Altered barrier function in the cells from people with AUD was associated with impaired colocalization of two proteins—claudin-7 and zonula occludens-1—which indicates disorganized tight junctions.^30^ This suggests that the tight junction disorganization seen in individuals with AUD may contribute to or worsen lung disease progression. The impact of alcohol misuse on SARS-CoV-2 infection is further reviewed in later sections. Taken together, these studies suggest that alcohol misuse primes airway epithelial cells for increased barrier dysfunction, rendering the lungs susceptible, in part, to secondary infectious damage.

Human airway epithelial cells obtained from people with and without AUD also have been evaluated using mRNA microarrays. The cells from individuals with AUD exhibited approximately 520 differentially expressed genes compared with people without AUD, including genes for ribosomal proteins and genes involved in protein folding, even when adjusting for smoking status.^31^ Further, enrichment analyses indicated significant differential expression of 24 gene ontology pathways (i.e., biological processes, cellular components, or molecular functions) in epithelial cells isolated from individuals with and without AUD, including those implicated in protein targeting to the membrane and viral gene expression.^31^ This suggests that alcohol can alter the transcriptional profile of airway epithelial cells, thus predisposing them to increased barrier disruption; however, more data are needed to confirm these observations.

#### Effects on mucociliary clearance

Chronic and acute ethanol exposure are known to alter airway mucociliary clearance, but specific effects depend upon the dose and duration of ethanol exposure. For example, several studies have demonstrated that low concentrations of ethanol (2 to 20 mM) or short ethanol exposure (1 to 6 hours) stimulated ciliary beat frequency (CBF).^32^–^34^ The ability of cilia to increase their beat frequency in response to stimulation is critical for optimal mucociliary clearance of inhaled particles and debris. This increase in CBF was dependent on airway epithelial nitric oxide production and on the activity of the two enzymes cyclic AMP-dependent protein kinase A (PKA) and cyclic GMP-dependent protein kinase G (PKG) in ciliated cells.^32^–^34^ With greater alcohol exposure (e.g., in people with alcohol misuse), lung clearance is generally thought to be impaired; however, more detailed analyses based on different drinking patterns have demonstrated that dose and duration of alcohol exposure have a large effect on mucociliary clearance. Specifically, subsequent experiments demonstrated that ethanol-induced CBF stimulation in vitro is transient and is followed by a rapid induction of ethanol-sensitive phosphodiesterase and downregulation of PKA in ciliated cells.^35^,^36^ This suggests that if bacterial aspiration occurs, cilia are not effectively stimulated due to alcohol-mediated downregulation of CBF. These findings have also been replicated in in vivo animal models. Specifically, ethanol-fed rats and mice exhibited slowed CBF and desensitization of airway PKA activity.^32^,^37^ Bacterial clearance was also impaired by ethanol and correlated with the degree of cilia desensitization.^38^

Alcohol’s effects on CBF have also been investigated using beta-agonists, which can stimulate CBF independent of the presence of particles or pathogens. Studies found that the ability to increase CBF following beta-agonist stimulation was significantly decreased after prolonged ethanol exposure. This prolonged ethanol exposure led to activation of protein phosphatase 1 (PP1) and blockage of cAMP-dependent cilia beating.^39^ Further, bovine cilia axonemes (i.e., the bundles of microtubules and associated proteins that form the core of cilia in eukaryotic cells) exposed to ethanol exhibited a marked increase in the S-nitrosylation of key cilia regulatory proteins. Specifically, ethanol exposure led to a marked increase in the S-nitrosylation of PP1, which was associated with PP1 activation and subsequent dysfunction of axoneme motility.^40^ In experimental animal models using bronchial alveolar lavage (BAL; a technique to collect cells and other materials from the airways and alveoli), S-nitrosothiol levels and PP1 activity were increased in ethanol-fed mice and were dependent on nitric oxide synthase 3. Similarly, treatment with S-nitrosoglutathione led to PP1-dependent CBF desensitization in mouse tracheal rings, cultured cells, and isolated cilia obtained from ethanol-naive wild-type mice.^41^ Finally, isolated axonemes exposed to a higher dose of alcohol (100 mM ethanol) for 1 hour exhibited no increase in CBF following cAMP stimulation. However, following 6 hours of exposure of axonemes to ethanol, the cAMP-mediated increase in CBF was restored to control levels. Treatment with thioredoxin—a potent antioxidant that reduces oxidized cysteine residues and cleaves disulfide bonds—reversed ciliary dysfunction in axonemes exposed to ethanol.^42^ This suggests that oxidative stress following ethanol exposure significantly impairs ciliary function. Taken together, these data suggest that redox modulation by ethanol is a key mechanism by which alcohol misuse impairs ciliary mechanisms.

#### Effects on immune cell function

As discussed in more detail below, chronic alcohol consumption significantly affects both innate and adaptive immune responses in the lungs. For example, ethanol consumption delayed tissue recruitment of neutrophils during infection and prevented their clearance following the resolution of injury.^5^ Ethanol also impaired the pathogen-clearing and immune responses of alveolar macrophages^9^,^11^,^12^ and decreased the number and function of dendritic cells.^13^–^15^ Finally, the number of lymphocytes circulating in the blood or recruited to the lungs was significantly decreased in ethanol-fed animals.^9^,^12^

##### Effects on neutrophils

Studies in rodents with pathogens, such as *Streptococcus pneumoniae*^43^ and in humans with blood alcohol concentrations as low as 0.10%^44^ demonstrated that ethanol impaired neutrophil recruitment to infected sites (i.e., the lung). Ethanol also delayed neutrophil clearance from the lungs, prolonging their presence postinfection.^7^,^43^ Additionally, ethanol may disrupt neutrophil phagocytosis and pathogen killing; however, the findings were inconsistent, with some studies showing impaired function, while others showed no effect, possibly due to differences in bacterial strains.^7^,^43^,^45^–^48^ Ethanol further suppressed neutrophil production in the bone marrow by interfering with granulocyte colony-stimulating factor (G-CSF; a key cytokine that drives neutrophil generation and function), leading to fewer neutrophils during infections.^49^,^50^ Pretreatment with G-CSF mitigated these deficits, enhancing neutrophil recruitment, bacterial killing, and survival in ethanol-consuming rats infected with *Klebsiella pneumoniae*. 51

##### Effects on macrophages

Ethanol-mediated impact on macrophage function is driven by multiple factors,^52^ including oxidative stress and zinc deficiency. Both of these were increased in the alveolar space after ethanol exposure.^52^,^53^ Specifically, chronic ethanol consumption upregulated nicotinamide adenine dinucleotide phosphate (NADPH) oxidase enzymes, which can produce harmful reactive oxygen species (ROS).^54^ Ethanol consumption also interfered with the nuclear factor E2-related factor 2-antioxidant response element pathway,^55^ reducing the ability of the macrophages to combat oxidative stress. Additionally, in human studies, alcohol induced zinc deficiency within alveolar spaces and downregulated GM-CSF receptors,^56^ both of which are vital for macrophage function. Restoration strategies, such as zinc supplementation and enhancing GM-CSF signaling, reversed these effects by improving redox balance, boosting immune gene expression, and restoring macrophage function.^55^,^57^

In vitro ethanol exposure of alveolar macrophages induced mitochondrial oxidative stress by increasing ROS production and depleting mitochondrial glutathione.^58^ Ethanol-induced TGFbeta1 and ROS production were associated with alveolar macrophage dysfunction and alternative macrophage activation, further impairing immune responses in the lungs of ethanol-exposed animals.^59^ Additionally, unstimulated alveolar macrophages from individuals with AUD produced greater levels of the proinflammatory cytokines interferon gamma and interleukin-(IL-) 6 than cells from individuals without AUD, suggesting a hyperinflammatory phenotype before stimulation.^60^ However, following culture with heat-killed *S. pneumoniae* protein, alveolar macrophages and peripheral blood mononuclear cells obtained both from people with AUD and people without AUD exhibited a dose- and time-dependent increase in proinflammatory cytokine production, with no differences between the two groups.^60^ Treatment with the potent antioxidant N-acetylcysteine reduced cytokine production from both cell types, except for IL-1beta, which remained elevated in cells obtained from people with AUD.^60^ These data suggest that alcohol misuse alters the basal inflammatory profile of macrophages and that mitigation of oxidative stress with N-acetylcysteine partially reverses proinflammatory cytokine secretion in macrophages obtained from individuals with AUD.

##### Effects on dendritic cells

Dendritic cells are key antigen-presenting cells in the immune system. Chronic alcohol exposure reduced the ability of these cells to present antigens effectively, thereby weakening T-cell activation and adaptive immune responses in the lungs. Specifically, ethanol consumption decreased the expression of molecules on the dendritic cell surface needed to stimulate a T-cell response, such as cluster of differentiation 80 (CD80), CD86, and major histocompatibility complex class II molecules. This decreased expression inhibited dendritic cell maturation and impaired the initiation of immune responses.^14^,^15^,^61^ Ethanol consumption also shifted the cytokine profiles of dendritic cells, often reducing proinflammatory IL-12 while increasing anti-inflammatory IL-10, thus promoting immune tolerance over activation and increasing infection susceptibility.^13^ Moreover, ethanol-fed mice exhibited a decreased total number of dendritic cells in the lungs following influenza virus infection.^62^ Pulmonary dendritic cells from ethanol-treated mice (20% EtOH in the water for 4 to 16 weeks) also failed to upregulate CD40 and CD80 expression in response to stimulation of toll-like receptors on their surface by antigen binding.^63^ Lack of CD40 and CD80 expression significantly impaired the dendritic cells’ ability to stimulate T-cell activation and differentiation.^64^ Thus, ethanol impaired the ability of dendritic cells to induce a robust adaptive immune response to infectious challenge. Finally, ethanol-feeding compromised dendritic cell migration to lymph nodes, hindering their ability to bridge innate and adaptive immunity in lung infections.^63^,^65^

##### Effects on lymphocytes

Ethanol impairs lymphocyte recruitment to the lungs in infected animals.^66^–^68^ Thus, ethanol feeding in mice led to marked dysregulation of natural killer cell effector function and pulmonary recruitment via increased TGF-beta signaling and suppression of aryl hydrocarbon receptor signaling.^66^ Natural killer cells isolated from ethanol-fed mice had a reduced ability to kill bacterial pathogens (e.g., *K. pneumoniae)*. Likewise, ethanol significantly altered the cells’ migratory capacity in response to various chemokines. Thus, natural killer cells isolated from ethanol-fed mice exhibited preferential migration in response to chemokines binding to the CXCR3 receptor but reduced migration in response to chemokines binding to the CCR2, CXCR4, and CX3CR1 receptors.^66^

Similar to ethanol’s effect on natural killer cells, chronic ethanol exposure induced substantial changes in T-cell immunity. Exposure to chronic ethanol resulted in T-cell activation characterized by increased expression of CD69, CD25, and CD44.^69^,^70^ In contrast, T-cells both from humans who misuse alcohol and from chronic ethanol-fed rodents exhibited a reduced ability to proliferate and could not be rescued by the addition of IL-2.^71^ Chronic ethanol exposure also affected the type of T-cell response to a pathogen—that is, whether a Th1 response occurred that enhances cellular immune responses, maximizes the killing efficacy of macrophages, and promotes proliferation of CD8^+^ T-cells, or a Th2 response that promotes B-cell proliferation and antibody class switching. Thus, infection of chronic ethanol-fed mice with *K. pneumoniae*, which normally generates a Th1 response, instead generated a profound Th2 response and IL-10 production within the lungs, and neutralization of the IL-10 response improved bacterial clearance.^12^ These data suggest that alcohol misuse shifts the T-cell phenotype and activation state to a nonproliferative inflammatory condition with significantly reduced ability to respond to infectious insult. Finally, chronic ethanol ingestion induced the accumulation of group 2 innate lymphoid cells (a type of immune cells crucial for immunity against certain parasites), reduced the production of the neuropeptide calcitonin gene-related peptide, and markedly suppressed ethanol-driven type 2 cytokine signaling, all of which contributed to increased lung inflammation and fibrosis in rodent models.^72^

#### Other mechanisms

Alcohol’s impacts on the lungs are multi-factorial, and many mechanisms remain to be investigated. However, in addition to the effects described above there are well-documented increases in alveolar oxidative stress^53^,^58^,^73^–^77^ and decreased antioxidant levels,^54^,^55^,^59^,^78^,^79^ impairment of surfactant protein D (SP-D) structure and function,^80^–^82^ reduced secretion of antimicrobial peptides,^83^–^85^ impaired transport of IgA into alveolar space,^86^ changes to the metabolic profile,^87^–^89^ and alterations in the pulmonary microbiota.^90^ These mechanisms, all of which likely significantly contribute to lung injury following alcohol consumption, are described below.

##### Oxidative stress and antioxidants

Chronic alcohol misuse compromises lung function through mechanisms related to increased oxidative stress, such as depletion of the antioxidant glutathione and increased ROS production. For example, as observed in alveolar macrophages, chronic ethanol exposure also inhibited the nuclear factor E2-related factor 2-antioxidant response element pathway in alveolar epithelial cells. This reduced antioxidant gene expression and exacerbated oxidative stress, leading to epithelial cell dysfunction.^73^ Similarly, co-exposure of rats to ethanol and tobacco smoke independently and synergistically increased oxidative stress, with elevated lipid peroxidation and reduced antioxidant enzyme activity, all of which amplified lung tissue damage.^74^ Subsequent clinical studies that investigated the relationship between airway antioxidant levels, AUD, and cigarette smoking found that AUD was significantly associated with reduced airway glutathione and increased oxidative stress, both of which were exacerbated by smoking.^79^ Similarly, levels of uric acid (a “danger molecule,” or damage-associated molecular pattern) in epithelial-lining fluid and activity of the enzyme xanthine oxidoreductase (which generates ROS) were substantially elevated in individuals with AUD.^91^ Xanthine oxidoreductase protein levels were also associated with increased cyclooxygenase 2 (COX-2) expression.^91^ Uric acid and ROS activated several proinflammatory pathways, including COX-2, suggesting that alcohol-mediated generation of these byproducts increased pulmonary inflammation, in part, via COX-2 signaling. Likewise, increased expression of another protein complex involved in the inflammatory response—NLR family pyrin domain containing 3 (NLRP3) inflammasome components—was sustained in LPS-stimulated airway cells obtained by BAL from individuals with AUD, and correlated with increased levels of IL-1-beta in BAL fluid.^91^ A marked increase in lung tissue oxidative stress also was observed in long-term alcohol-exposed rodents, as measured by increased lipid peroxidation, protein oxidation, and reduced antioxidant enzyme levels, resulting in structural and functional lung damage.^76^ More recently, ethanol-induced inflammation and oxidative stress in macrophages were demonstrated to be significantly mitigated by astaxanthin (a keto-carotenoid metabolite of zeaxanthin and canthaxanthin), which is thought to work by modulating enzymes involved in the regulation of gene expression.^78^ This suggests that mitigation of inflammation and oxidative stress in the lungs following alcohol misuse may be a viable therapeutic target for the prevention of alcohol-associated respiratory disease.

##### Surfactant protein D

Malondialdehyde and acetaldehyde are formed during ethanol metabolism and tobacco pyrolysis.^92^ The two compounds can form a malondialdehyde-acetaldehyde (MAA) adduct—a stable protein adduct (a compound formed when a reactive molecule covalently binds to a protein altering its structure and function) that is known to be immunogenic, pro-fibrotic, and proinflammatory.^93^ MAA adduction affects SP-D, a critical component of pulmonary innate immunity, and this process plays a major role in lung innate defense. Specifically, MAA adduction significantly reduced SP-D’s ability to bind to the SARS-CoV-2 spike protein, impairing viral neutralization.^80^ These data expanded on prior work demonstrating that MAA adduction altered SP-D’s structure, leading to dysfunctional immune responses, including reduced pathogen clearance and increased inflammation.^81^

These findings have been further extended to investigate the impacts of MAA adducts on lung health. Specifically, the use of tobacco or alcohol and the subsequent formation of MAA protein adducts were shown to induce lung injury characterized by inflammation and tissue damage, likely due to the activation of proinflammatory pathways.^82^ Other studies in mice lacking functional scavenger receptor A (SR-A1 knockout mice) found decreased BAL cellularity, influx of neutrophils, CXCL1 BAL levels, and MAA adduct staining in the lung epithelium.^94^ SR-A1 is mainly found on myeloid cells and functions as a pathogen-associated molecular pattern receptor and damage-associated molecular pattern. Activation of this pathway led to proinflammatory cytokine production.^95^ This suggests that immune recognition of MAA adducts via SR-A1 is a critical driver of the detrimental effects of MAA adducts on the lungs. Furthermore, people with AUD who smoke had increased MAA adduct formation, which decreased immunoglobulin A (IgA) transport across airway epithelium, thereby weakening mucosal immunity.^86^ Finally, MAA adducts were exclusively detected in the lungs of individuals who smoke and misuse alcohol and were associated with systemic anti-MAA antibodies,^92^ suggesting a chronic and detrimental immune response to these adducts.

##### Antimicrobial peptides

Alcohol also negatively impacts pulmonary antimicrobial defenses. Ethanol exposure depleted pulmonary vitamin D and the production of the antimicrobial peptide cathelicidin. However, treatment with diallyl disulfide (a garlic-derived compound) mitigated the effect of ethanol treatment.^83^ Acute ethanol exposure also impaired the clearance of methicillin-resistant *Staphylococcus aureus* in the lung by reducing epithelial production of Reg3gamma, an antimicrobial peptide critical for bacterial killing.^84^ Similarly, individuals with AUD exhibited reduced antimicrobial proteins in epithelial lining fluid, which was associated with compromised antipneumococcal activity and increased infection risk.^85^

##### Metabolic profile

The effects of alcohol-related metabolic dysregulation in alveolar macrophages and other immune cells also have become an area of interest. Ethanol treatment led to marked lipid dysregulation in alveolar macrophages, which significantly impaired the glycolytic response to LPS, thus weakening the cells’ ability to mount effective immune responses.^87^ Ethanol exposure also inhibited AMP-activated protein kinase signaling and phagocytosis in alveolar macrophages, with ethanol metabolism playing a central role in these impairments.^88^ Additionally, ethanol exposure led to marked increases in mitochondrial DNA (mtDNA) damage.^96^ Specifically, exosomes isolated from ethanol-exposed mouse alveolar macrophages increased mtDNA damage and barrier dysfunction in a mouse alveolar epithelial cell line. Likewise, exosomes from the ethanol-exposed alveolar epithelial cells led to mtDNA damage and phagocytic dysfunction in alveolar macrophages.^96^ This suggests that exosomes enriched with alcohol-associated mtDNA damage may contribute to injurious cross-talk between the alveolar epithelium and macrophages.^96^

##### Pulmonary microbiota

Finally, studies found that the spatial distribution of the microbiome (i.e., biogeography) throughout the respiratory tract was significantly altered in individuals with AUD.^90^ Specifically, people with AUD had marked increases in the mean species diversity in a given location characterized by outgrowth of Gram-negative bacteria and loss of biogeography of the microbial communities in the upper and lower airways.^90^ Pulmonary dysbiosis (a change in the normal commensal microbial communities that is associated with or leads to disease) may likely exacerbate lung inflammation and impair immune responses, thus increasing susceptibility to infections. However, further studies are needed to verify the effects of pulmonary dysbiosis on alcohol-associated types of pneumonia or injury.

### Alcohol Misuse and Pneumonia

It is widely accepted that AUD is an independent risk factor for a wide variety of lung infections, including pneumonia, and increases morbidity and mortality for people with pneumonia. A recent meta-analysis demonstrated an approximately 8% increase in the relative risk of community-acquired pneumonia for every 10 to 20 grams of alcohol consumed per day.^3^ The following sections will review the impacts of alcohol on pneumonia development and host defense against bacterial, fungal, and viral pathogens.

#### Bacterial pneumonia

Ethanol ingestion has been shown to increase pneumonia severity and mortality following lung infection with *K. pneumoniae*,^9^,^12^,^48^,^66^, ^68^,^97^–^101^ *Mycobacterium tuberculosis*,^102^ *S. pneumoniae*,^7^,^38^,^43^,^49^,^50^,^60^,^97^,^103^–^106^ and *Pseudomonas aeruginosa*. 107 Although *S. pneumoniae* was the most common cause of bacterial pneumonia in the general population as well as in individuals who misuse alcohol, *K. pneumoniae* infections were more severe and resulted in higher mortality in people with AUD.^108^,^109^ Altogether, respiratory bacterial infections in the context of chronic alcohol ingestion suggest that alcohol increases overall morbidity and mortality by increasing and sustaining the pathogen burden via the multi-factorial impacts on the lungs that are described above.

Recently, the negative impact of alcohol consumption on the multi-organ cross-talk along the gut-lung or gut-liver-lung axis has been demonstrated to be one of the drivers of alcohol-associated bacterial pneumonia. Specifically, mice transplanted with fecal microbiota from patients with AUD showed a higher susceptibility to pneumonia induced by *K. pneumoniae* or *S. pneumoniae* compared to mice colonized with fecal microbiota from healthy individuals without AUD.^97^ Ethanol-associated dysbiosis due to transfer of microbiota from ethanol-fed mice also led to a higher rate of *K. pneumoniae* infection independent of direct ethanol consumption in mice. 67,98 *Klebsiella pneumoniae* burden was associated with an increase in inflammatory cytokines in the lung and a decrease in the number of immune cells in the lung, both of which were partly driven by impairments in aryl hydrocarbon receptor signaling. 67,98 Administration of a probiotic cocktail or the microbial metabolite indole reduced the risk for ethanol-associated pneumonia.^98^

Other studies found an additive effect on the gastrointestinal microbiota-associated increases in susceptibility to lung infections in ethanol-fed mice infected with HIV.^105^ More precisely, mice colonized with the microbiota from ethanol-fed mice infected with HIV had a higher *S. pneumoniae* lung burden when compared to mice colonized with an HIV-associated microbiota or an ethanol-associated microbiota.^105^ These findings were further supported by clinical data, which demonstrated that active alcohol use was associated with increased pneumonia severity in persons living with HIV.^110^ Alcohol’s effect on pneumonia severity differed by sex, with increased pneumonia severity in women with HIV who misuse alcohol.^110^

Ethanol-associated changes in the gastrointestinal bacterial taxa and metabolic processes were also associated with increased levels of neutrophils (Ly6G cells), the cytokine lipocalin-2, and the enzyme myeloperoxidase in the lungs. These components are part of the normal host response to bacterial pneumonia; however, excess or uncontrolled production may lead to an exacerbated inflammatory environment in the lungs.^111^ Supplementation with tributyrin (an agonist for butyrate-stimulated pathways) during ethanol exposure restored both the number of Ly6G+ cells and lipocalin-2 levels in the lungs to levels seen in control-fed mice.^111^ Finally, ethanol exposure increased the number of activated macrophages and neutrophils in the lungs following bacterial challenge with *P. aeruginosa*, all of which correlated with ethanol-induced changes in the gut microbiome and immune environment.^107^

#### Fungal pneumonia

Studies on alcohol’s impact on fungal infections are perhaps the most ill-defined, and few studies have addressed these questions. In addition, the clinical/epidemiological data to support an association between alcohol misuse and fungal pneumonia is lacking and represents a clear avenue for further exploration and study.

In mice, intraperitoneal acute ethanol exposure prior to *Pneumocystis carinii* challenge significantly decreased tumor necrosis factor (TNF) activity and the number of certain white blood cells (polymorphonuclear leukocytes) in BAL.^8^ Further, chronic ethanol consumption (4% EtOH liquid diet for 7 weeks) increased the severity of *P. carinii* infection, as *P. carinii* was present in more than 60% of the ethanol-fed mice but was not detected in the control animals. Higher pathogen burden was associated with a higher number of polymorphonuclear leukocytes in ethanol-fed mice with persistent infection.^8^ Similarly, chronic ethanol consumption led to increased mortality rates and a higher pathogen burden after *Aspergillus fumigatus* challenge.^112^ Increased burden and mortality were associated with a significant reduction in pulmonary neutrophil recruitment, characterized by a decrease in leukocyte adhesion and rolling after ethanol consumption.^112^ Additionally, bone marrow-derived neutrophils from ethanol-fed mice showed lower fungal clearance and defective ROS production.^112^ Finally, the promoter of the IL-6 gene in bone marrow-derived macrophages from ethanol-chimeric mice (i.e., mice that received a bone marrow transplant from ethanol-fed mice) exhibited a reduction in histone methylation (i.e., H3K27me3 enrichment), which was associated with a persistent inflammatory response in lung tissue; increased lung damage; neutrophil accumulation; and IL-6, TNF, and CXCL2 production.^113^ Neutrophil-mediated killing and phagocytosis of *A. fumigatus* were also significantly lower.^113^ These data suggest that alcohol misuse likely contributes to fungal pneumonia via increased lung inflammation, neutrophil accumulation, and alterations to chromatin remodeling. However, human clinical studies, as well as epidemiological studies, are needed to better define the clinical relevance of alcohol misuse and fungal pneumonia.

#### COVID-19 and other viral pneumonia

Studies on alcohol’s impact on viral infections are likewise limited. Existing research in mice found that binge ethanol consumption before influenza infection increased mortality, sustained high viral titers, and prolonged virus presence in the lungs.^114^ Further, chronic ethanol consumption in mice significantly increased morbidity and mortality from a low-dose influenza A infection, with 50% of ethanol-fed mice dying compared to about 1% of controls.^62^ The ethanol-fed mice showed greater weight loss, higher viral titers, and severe lung pathology, including increased neutrophilia and edema.^62^ Given the continued public health burden associated with influenza each year, research that investigates the impact of alcohol misuse on influenza susceptibility, vaccine efficacy, or virus transmission is sorely needed, especially with modern advancements and novel technologies for the study of viral diseases.

Chronic ethanol consumption also increased susceptibility to and severity of respiratory syncytial virus (RSV) infections in mice. Ethanol-exposed animals showed prolonged pulmonary inflammation, neutrophilia, edema, hemorrhage, and delayed viral clearance by at least 5 days compared to control mice.^65^ Additional studies are needed to better define the clinical relevance of co-occurring alcohol misuse and RSV infection.

Interest in the impact of alcohol on viral pneumonia has seen a resurgence since the beginning of the COVID-19 pandemic. Several observational and review analyses suggest that AUD and alcohol misuse could be associated with increased COVID-19 severity.^115^–^121^ However, only a few studies have demonstrated a significant impact of alcohol misuse and AUD on COVID-19 severity, identifying them as independent risk factors for worse clinical outcomes, such as higher rates of hospitalization, complications, and all-cause mortality in COVID-19 patients.^122^,^123^ These effects are likely driven by mechanisms such as immune suppression and chronic inflammation. A few mechanistic studies have begun to elucidate the mechanism by which alcohol impairs host defense against SARS-CoV-2. For example, a recent study demonstrated that MAA adducts from ethanol metabolism impaired the ability of surfactant protein D to neutralize SARS-CoV-2, increasing susceptibility to severe infection.^80^ In addition, bronchial epithelial cells from individuals with AUD showed a significant decrease in barrier function post SARS-CoV-2 infection, whereas the barrier function of cells obtained from individuals without AUD was enhanced post SARS-CoV-2 infection.^30^ Further, SARS-CoV-2-infected bronchial cells from subjects with an AUD had enhanced secretion of multiple proinflammatory cytokines, including TNF-alpha, IL-1beta, and interferon-gamma.^30^ Cytokine production in response to infection is a normal physiological response. However, an exacerbated cytokine response, as seen in the cells from individuals with AUD, may increase the likelihood of developing an extreme immune response where the body releases too many cytokines (cytokine storm), leading to severe symptoms, organ failure, or death. Finally, individuals with substance use disorders, including AUD, face heightened risks of developing lung infections due to social vulnerabilities (e.g., low social support, housing instability, or socioeconomic adversity) and compromised health.^115^

### Alcohol Misuse and Airway Disease

Alcohol misuse has also been identified as an independent risk factor for the development of airway disease, specifically for ARDS.^17^ However, the impact of alcohol on lung airway function is dependent on the concentration, duration and route of exposure. As previously discussed, chronic alcohol consumption can impair lung immune response and physiological function, ultimately making individuals susceptible to comorbid infections. The following sections will review the airway diseases that may occur as consequences of alcohol misuse.

#### ARDS

ARDS is two to four times more common in individuals with AUD than in those without AUD.^124^ In fact, a meta-analysis showed that any measure of high, relative to low, alcohol consumption was associated with a significantly increased risk of ARDS. Further, sensitivity analyses indicated that this association was attributable primarily to a history of alcohol misuse.^125^ Chronic alcohol consumption increases susceptibility to ARDS through multiple mechanisms. Research demonstrated that alcohol impaired alveolar epithelial and endothelial cell barrier properties. At baseline, pulmonary edema did not develop due to compensatory mechanisms; however, these compensatory mechanisms failed during inflammatory challenges, leading to alveolar flooding.^22^ Alcohol misuse was also associated with a fivefold increase in activated TGF-beta1 following endotoxemia, which directly impaired epithelial integrity.^26^,^126^ Additionally, alcohol induced oxidative stress by depleting glutathione and upregulating NADPH oxidase, which produced ROS.^52^–^55^,^57^ All of these effects predispose the lung to the development of ARDS following exposure to an injurious insult (i.e., trauma, sepsis/septic shock, pneumonia). More recently, acute ethanol consumption was demonstrated to worsen sepsis-induced lung injury in mice by activating the sphingosine kinase 1 (SphK1)/sphingosine-1-phosphate (S1P)/S1P receptor 1 (S1PR1) signaling pathway.^127^ Specifically, acute ethanol exposure led to increased pulmonary vascular permeability, neutrophil infiltration, inflammation, oxidative stress, and cell apoptosis. Furthermore, treatment with the SphK1 inhibitor PF-543 significantly alleviated the effects of sepsis-induced lung injury.^127^ Similarly, LPS-mediated acute lung injury in ethanol-fed mice was significantly reduced in animals treated with mitoquinone, a mitochondria-targeted antioxidant.^128^ Mitoquinone treatment significantly reduced lung inflammation and tissue damage by inhibiting excessive removal of mitochondria (mitophagy) and suppressing activation of the NLRP3 inflammasome.^128^ These data suggest that targeting the SphK1/S1P/S1PR1 pathway or preserving mitochondrial integrity could represent promising new therapeutic strategies for managing sepsis-related lung injury in the context of acute ethanol intoxication.

#### Other airway diseases

Alcohol misuse has also been linked to other diseases of the airway, such as chronic obstructive pulmonary disease (COPD), asthma, lung fibrosis, and changes in bronchial motor tone.

##### COPD

A large prospective cohort study involving more than 44,000 Swedish men found that moderate alcohol consumption (i.e., seven to 14 drinks per week), particularly beer and wine, was associated with a reduced risk of developing COPD. Furthermore, a J-shaped association for total grams of ethanol consumed per day and for beer consumption, and a U-shaped association for wine consumption was observed, indicating that moderate consumption may be protective, while higher intake levels do not confer additional benefits.^129^ Similarly, a study analyzing data from middle-aged men in Finland, Italy, and the Netherlands over a 20-year period found a U-shaped relationship between alcohol consumption and COPD mortality. People with light alcohol consumption (i.e., more than one drink per week but no more than three drinks per day) had the lowest risk of COPD mortality compared to nondrinkers (i.e., one drink or less per week) and people who drank heavily (i.e., more than nine drinks per day). Pulmonary function tests (forced expiratory volume in 1 second [FEV1] or 0.75 seconds [FEV0.75]) were also higher in people with light drinking compared to people with no alcohol consumption.^130^ A cohort study of more than 126,000 individuals found that heavy alcohol consumption (more than six drinks per day) was associated with a significantly increased risk of hospitalization for COPD. People with moderate consumption (one to two drinks per day) had a lower risk compared to those who had never consumed alcohol, but the protective effect was not observed in people with heavy drinking.^131^ Additionally, a study of 30,503 veterans found that individuals who had scores of 6 or more on the Alcohol Use Disorders Identification Test—Consumption (AUDIT-C), scores of 2 or more on the CAGE test, or who reported binge drinking (six or more drinks on an occasion) at least weekly were at an increased risk of COPD exacerbation in an age-adjusted analysis. However, after adjustment for tobacco use, the association between alcohol consumption and increased risk of COPD exacerbation was no longer evident.^132^ Finally, a study examining the impact of AUD on patients with asthma and COPD found that those with AUD were more likely to present with respiratory failure, require mechanical ventilation, have longer hospital stays, and have higher health care costs than those without AUD, which suggests that treating AUD in this patient population may improve disease outcomes and reduce health care utilization.^133^

##### Asthma

The relationship between alcohol consumption and asthma is complex. A study involving more than 19,000 Danish twins found a U-shaped association between alcohol intake and adult-onset asthma risk. Moderate alcohol consumption (1 to 6 units/week) was associated with the lowest risk, while both people who rarely or never drank and those with heavy daily drinking had higher risks.^134^ Alcohol also influenced immune responses that potentially affect asthma development.^135^ For example, alcohol may affect allergic sensitization and total levels of IgE, as well as lymphocyte subsets that drive a Th1/Th2 cytokine imbalance. Alcohol consumption can also influence bronchial motor tone. In patients with alcohol-induced asthma, inhalation of ethanol reduced nonspecific bronchial responsiveness, suggesting a bronchodilatory effect.^136^ However, prolonged and heavy exposure to alcohol impaired mucociliary clearance and may complicate asthma management.^4^ While chronic ethanol exposure in mice did not result in marked changes in lung function, ethanol-feeding plus gavage (binge) ethanol led to neutrophil accumulation in both the lung tissue and the BAL fluid, all of which was associated with airway hyper-responsiveness to inhaled methacholine (a medication that induces airway narrowing).^137^ These findings suggest that dose and duration may be critical factors in understanding the impacts of alcohol on bronchial motor tone.

##### Pulmonary fibrosis

Chronic alcohol consumption has been increasingly recognized as a contributing factor to pulmonary fibrosis, particularly in the context of acute lung injury. Experimental studies using murine models have demonstrated that chronic ethanol ingestion primes the lungs for an exaggerated fibrotic response following injury. For example, ethanol consumption enhanced bleomycin-induced lung fibrosis and inflammation by regulating type 2 innate immune responses, particularly involving group 2 innate lymphoid cells.^72^ Alcohol also reduced the production of the neuropeptide calcitonin gene-related peptide that regulates inflammation.^72^ These changes likely contribute to increased lung inflammation and fibrosis. Another study demonstrated that chronic ethanol ingestion promoted an aberrant fibrotic response to bleomycin-induced acute lung injury in mice, which was associated with increased expression and activation of the pro-fibrotic cytokine TGFbeta1 and increased collagen deposition.^138^ Compared with control mice, animals subjected to 8 weeks of ethanol feeding exhibited a 75% increase in lung collagen deposition and a 120% elevation in active TGF-beta1 levels in BAL fluid after bleomycin-induced lung injury.^138^ Supplementation with the glutathione precursor *S*-adenosylmethionine attenuated these fibrotic responses by reducing TGF-beta1 expression and collagen accumulation, indicating a potential therapeutic avenue for mitigating alcohol-induced pulmonary fibrosis.^138^ Further supporting these findings, chronic ethanol exposure has been shown to disrupt the balance of matrix remodeling enzymes in the lung. Thus, ethanol activated matrix metalloproteinases that are involved in the degradation of the extracellular matrix, particularly MMP-2 and MMP-9,^139^ which may further drive lung remodeling following ethanol exposure.

These studies collectively indicate that, whereas moderate alcohol consumption may have a protective effect against COPD and asthma, heavy drinking and alcohol misuse are associated with worsened outcomes and increased health care burdens. The data also suggest that although acute alcohol exposure may have bronchodilatory effects, chronic misuse can lead to detrimental changes in bronchial motor tone and overall airway function.

## Conclusions and Future Directions

Chronic alcohol misuse, particularly AUD, significantly impairs lung health by increasing susceptibility to infections, such as bacterial, fungal, and viral pneumonias, and heightening the risk of ARDS and other airway diseases, including COPD, asthma, and pulmonary fibrosis. These effects stem from multifaceted mechanisms, including compromised mucociliary clearance, disrupted epithelial and endothelial barrier functions, and impaired innate and adaptive immune responses. Key findings regarding the impact of alcohol consumption include an 8% increase in risk of community-acquired pneumonia per 10 to 20 grams of daily alcohol consumption;^3^ a twofold to fourfold higher risk of ARDS in individuals with AUD;^17^ and exacerbated lung injury driven by oxidative stress,^73^ zinc deficiency,^52^,^53^ and altered signaling pathways such as TGF-beta1 and GM-CSF.^55^,^57^,^59^ Alcohol-induced changes in the gut-lung axis and pulmonary microbiota further amplify infection susceptibility.^67^,^98^ Although moderate alcohol consumption may offer protective effects against COPD^129^,^130^ and asthma,^134^ heavy drinking consistently worsens outcomes, increasing morbidity, mortality, and health care burdens; this suggests a U-shaped dosing effect of alcohol for these conditions, as well as for pulmonary fibrosis. This contrasts with the literature associating alcohol with a linear increase in the risk of developing ARDS and pneumonia. These potential differences may be due to the inherent nature of the different diseases, dose- and duration-dependent effects, or other underlying mechanisms not yet identified, leaving these areas primed for future investigations.

Despite these advancements in understanding alcohol’s effects on the lungs, several limitations in the field still exist. For example, studies that investigate dose- and duration-dependent effects of alcohol on the lung, sex-specific differences in lung responses, or the interaction of alcohol with other co-exposures/comorbidities, such as smoking and HIV are very sparse. In addition, well-defined observational and longitudinal human studies employing robust measures of alcohol use are limited. Future research could include mechanistic studies to elucidate the precise molecular pathways underlying alcohol-induced lung injury, particularly the role of gut-lung axis dysbiosis and its therapeutic modulation via probiotics or microbial metabolites. Longitudinal human studies could enhance understanding of dose- and duration-dependent effects of alcohol on lung epithelial barrier function and immune responses, especially in the context of viral infections, such as SARS-CoV-2. Additionally, analyses of targeted interventions, such as zinc supplementation, GM-CSF restoration, or treatment with glutathione precursors, such as *S*-adenosylmethionine, could support development of novel therapeutic strategies to mitigate alcohol-related lung damage. Investigating sex-specific differences in alcohol’simpact on lung health, as well as its interaction with co-occurring conditions, such as HIV, may provide insight into how to further refine clinical approaches. Finally, public health efforts that focus on integrating AUD treatment into respiratory care may help reduce the incidence and severity of alcohol-associated lung diseases and improve patient outcomes.

## Figures and Tables

**Figure 1:**
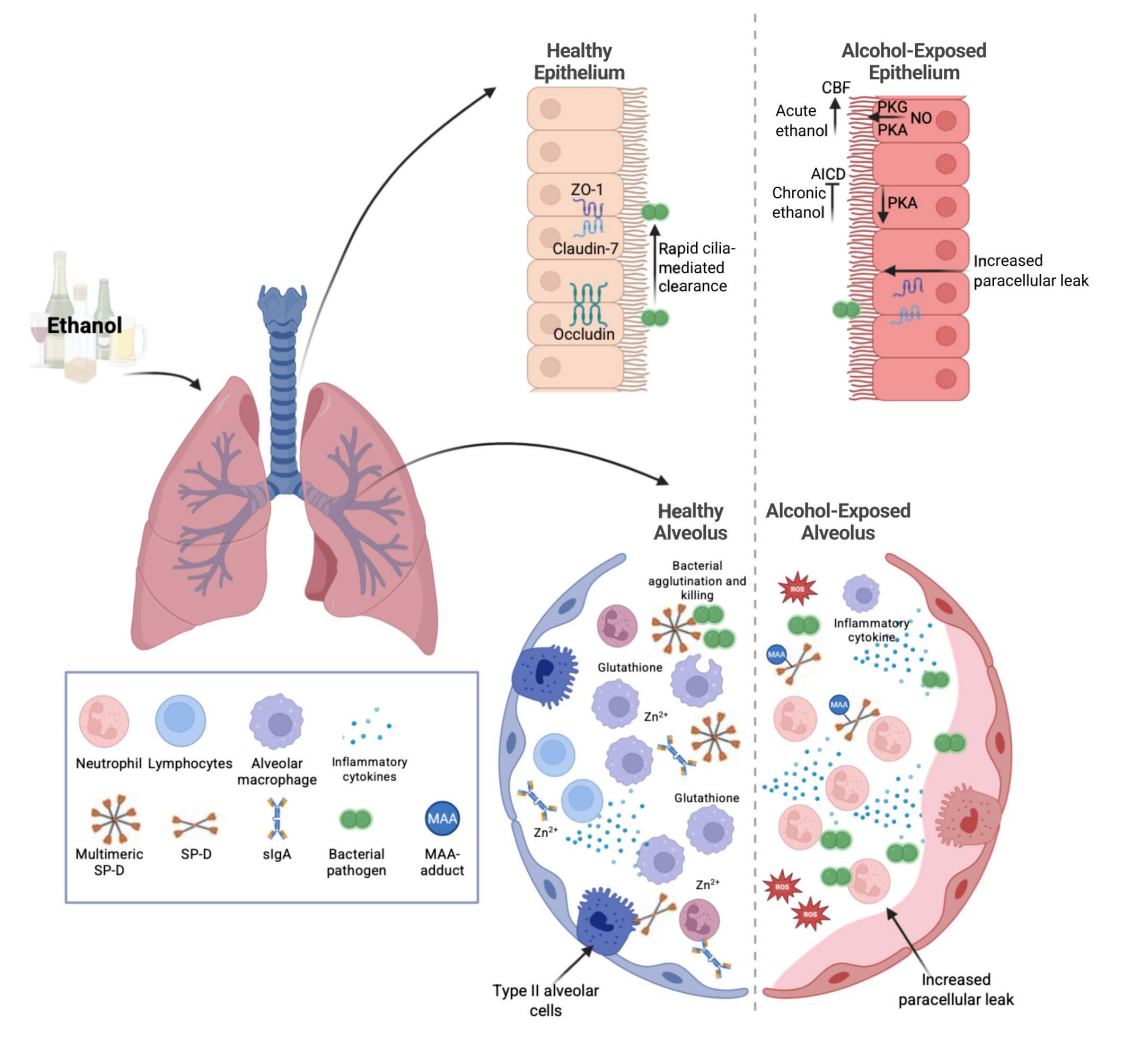
Ethanol’s effects on the lung. Ethanol leads to numerous changes in the lungs. These include impaired epithelial barrier function, mucociliary clearance, immune cell responses, and changes in soluble factors. Note: AICD, alcohol-induced ciliary dysfunction; CBF, cilia beat frequency; MAA, malondialdehyde-acetaldehyde; NO, nitric oxide; PKA, protein kinase A; PKG, protein kinase G; ROS, reactive oxygen species; sIgA, secreted immunoglobulin A; SP-D, surfactant protein D; Zn^2+^, zinc; ZO-1, zonula occludens-1. Source: Created in BioRender. Samuelson, D. (2025). https://BioRender.com/r6422ik.

**Table 1:** Key Impacts of Alcohol on the Lung

Lung Functions	Baseline Effects of Alcohol	Effects of Alcohol With Secondary Infection / Injury
**Lung Epithelial Barrier**	Loss of tight junctions Zonula occludens-1 Claudin-7 Increase in Na/K ATPase	Edema Increased leak Pathogen dissemination
**Mucociliary Clearance**	Acute alcohol Increased cilia beat frequency Chronic alcohol Alcohol-induced cilia dysfunction Altered PP1, PKA, and PKG signaling Increased S-nitrosothiol	Increased S-nitrosothiol Increased pathogen burden
**Lung Immune Cell Functions**	Impaired alveolar macrophage function Decreased lymphocytes and PMN Increase NADPH oxidases in alveolar macrophages	Impaired cell recruitment Loss of PMN clearance Impaired alveolar macrophage phagocytosis Impaired PMN and NK cell cytotoxicity Shifted Th1/Th2 profiles
**Soluble Factors**	Increased TGF-beta Decreased GM-CSF MAA-adducted proteins (SP-D) Increased ROS Decreased zinc Decreased glutathione	Impaired agglutination Increased inflammatory cytokines Increased ROS Loss of secreted IgA in the airway

*Note*: GM-CSF, granulocyte-macrophage colony-stimulating factor; IgA, immunoglobulin A; MAA, malondialdehyde-acetaldehyde; NADPH, nicotinamide adenine dinucleotide phosphate; Na/K-ATPase, sodium-potassium adenosine triphosphatase; NK, natural killer; PKA, protein kinase A; PKG, protein kinase G; PMN, polymorphonuclear leukocyte; PP1, protein phosphatase 1; ROS, reactive oxygen species; SP-D, surfactant protein D; TGF-beta, transforming growth factor beta; Th1, T helper cell type 1; Th2, T helper cell type 2.
